# Histopathological Analysis of Hysterectomy Specimens in a Tertiary Care Centre: A Retrospective Study

**DOI:** 10.7759/cureus.50497

**Published:** 2023-12-14

**Authors:** Rashmi Wankhade, Pratibha Dawande

**Affiliations:** 1 Pathology, Datta Meghe Medical College, Datta Meghe Institute of Higher Education and Research, Nagpur, IND

**Keywords:** hysterectomy, leiomyoma, follicular cyst, chronic cervicitis, adenomyosis

## Abstract

Introduction

The uterus is a crucial reproductive organ that is susceptible to the development of several non-neoplastic and neoplastic diseases in women, greatly increasing morbidity and mortality. Although there are various therapeutic options, hysterectomy is still a popular treatment option throughout the world. Abnormal uterine bleeding, pelvic pain, pelvic inflammatory disease (PID), prolapse of the uterus, adenomyosis, endometriosis, fibroids, gynecological malignancies, and obstetric problems that require hysterectomy, all samples must be examined histopathologically. Histopathological examination of the specimens obtained after hysterectomy is important for both diagnosis and treatment. The current work aimed to identify the various clinical indications, analyze the clinicopathological correlation in hysterectomy specimens, and analyze the patterns of lesions in hysterectomy specimens.

Materials and methods

This study was conducted in the Department of Pathology at the Datta Meghe Medical College, Wanadongari, Nagpur, from February 2022 to January 2023. All types of hysterectomy specimens received during this year were examined, and the tissues were processed and stained with H&E. Histopathological examination was performed, and various lesions in the hysterectomy specimens were examined. The study included all forms of hysterectomy, including abdominal, vaginal, laparoscopic, and total abdominal hysterectomy.

Results

An analysis of 110 cases of hysterectomy revealed that abdominal hysterectomy was the type of hysterectomy in 79 (71.82%) cases, with a maximum age range of 35 to 45 years (42.72%). The proliferative phase endometrium was the most common endometrial pathology, accounting for 43 (39.09%) cases, followed by the atrophic endometrium in 35 (31.82%) cases. Leiomyoma was the most prevalent myometrial lesion, accounting for 52 (47.28%) cases, followed by adenomyosis, accounting for 23 (20.91%) cases. Chronic cervicitis was the most common incidental finding in the hysterectomy samples, accounting for 85 (77.28%) cases. Follicular cysts, representing 22 (20%) cases, were the most common ovarian lesions, followed by serous cystadenoma in seven (6.37%) cases. Two cases of malignant tumors were noted: one case of endometrial carcinoma and one case of mucinous cystadenocarcinoma of the ovary. In most cases, ranging from 70% to 100%, the final histopathological diagnosis supports the preoperative clinical diagnosis.

Conclusion

Hysterectomy is the most common major gynecological surgery performed under elective conditions. Although histological studies and clinical diagnoses are closely correlated, several lesions, including chronic cervicitis and adenomyosis, were discovered incidentally. Therefore, every hysterectomy specimen must undergo a thorough histological investigation, even if it appears superficially normal, to confirm the diagnosis and improve postoperative care.

## Introduction

The female genital system is an intricate but intriguing structure that includes the external and internal genitalia, uterus, ovaries, fallopian tubes, and vagina. One of the most significant female reproductive organs is the uterus, which is also known as the womb and cervix. They are susceptible to both neoplastic and non-neoplastic diseases. The endometrium and myometrium, which make up the uterus, are constantly driven by hormones, inhabited by fetuses, and undergo a monthly loss of endometrial mucosa [1]. Along with cervix lesions, endometrial and corpus of the uterus are the most common causes of patient visits to gynecologists [2].

Despite a variety of treatment options, such as medication and conservative surgical techniques, hysterectomy remains the most common gynecological procedure performed globally [3]. The first partial hysterectomy was performed in Manchester, England, in 1843 by Charles Clay, and in 1929, the first total abdominal hysterectomy was performed [4]. Numerous conditions such as abnormal uterine bleeding, pelvic pain, pelvic inflammatory disease (PID), prolapse of the uterus, adenomyosis, endometriosis, fibroids, gynecological malignancies, and obstetric problems have been reported. Every hysterectomy sample must be examined histopathologically because histology is the only source of the final diagnosis [5]. Histopathological examination of the specimens obtained after hysterectomy is important for both diagnosis and treatment. The current work aimed to identify the various clinical indications, analyze the clinicopathological correlation in hysterectomy specimens, and analyze the patterns of lesions in hysterectomy specimens.

## Materials and methods

Study design

This is a hospital-based retrospective observational study.

Place and duration of study 

This study was carried out in the Pathology Department at Datta Meghe Medical College, Wanadongari, Nagpur, from February 2022 to January 2023.

Sample size

A total of 110 women who presented to the obstetrics and gynecology department of the Datta Meghe Medical College, Wanadongari, Nagpur, with a clinical diagnosis of female genital tract lesions, were enrolled in the study.

Inclusion criteria

The study included all forms of hysterectomy, including abdominal, vaginal, laparoscopic, and total abdominal hysterectomy, with or without unilateral or bilateral salpingectomy or salpingo-oophorectomy.

Exclusion criteria

Obstetric hysterectomy was the only exclusion criterion for this study.

Data collection 

The documentation included the patient's name, age, sex, clinical appearance, and differential diagnosis, along with a detailed clinical history and information from the gynecological request form.

Study procedure

The samples were gathered and sent to the histopathology section of the pathology department. From the patient's case report, a succinct summary of the pertinent clinical history and results was taken. The samples received were fixed with 10% neutral buffered formalin. The samples were grossly examined and sectioned. In case if required, representative sections from anomalous regions were also taken. H&E staining was performed on the sections after they had been embedded and processed. The slides were all examined under a microscope. Statistics were conducted after taking note of the results.

Ethical consideration

As this study was retrospective and observational rather than interventional, there were no risk variables. Information was collected from the patient's request form and histopathological findings of the pathology department. The appropriate authorization was obtained from the Institutional Ethics Committee (SMHRC/IEC/2022/12-15). The identities of patients and doctors were not noted.

Statistical analysis

The tabulated results were subjected to statistical analyses. Statistical analysis was used to determine the type of lesion, the incidence rate, and the percentage in each age group. After entering the data into Microsoft Excel (Microsoft Corporation, Redmond, Washington, United States), statistical analysis was performed.

## Results

This study included 110 patients. The age distribution of the hysterectomy specimens is presented in Table 1. Hysterectomies were performed in women aged between 25 and 75 years of age. The majority of cases, 47 (42.72%) of these 110 cases, occurred between the ages of 35 and 45 years, followed by 32 (29.09%) cases between the ages of 45 and 55 years. The least number of cases, four (3.64%), were between the ages of 65 and 75 years.

**Table 1:** Age distribution of hysterectomy specimens (N = 110)

Age group (years)	Number of cases (N)	Percentage (%)
25 – 35	14	12.72
35 – 45	47	42.72
45 – 55	32	29.09
55 – 65	13	11.81
65 – 75	4	3.64

According to Table 2, vaginal hysterectomy was the second most prevalent type of hysterectomy, with 31 cases (28.19%), while the most frequent type of hysterectomy performed was total abdominal hysterectomy with unilateral or bilateral salpingo-oophorectomy, which represented 79 cases (71.82%). The indications for hysterectomies range from irregular menstruation to possible pelvic malignancies. Table 2 shows the number of hysterectomy indications. Most of the patients had a fibroid uterus, which accounted for 33 (30%) cases, followed by uterovaginal prolapse, comprising 31 (28.19%) cases, and dysfunctional uterine bleeding, comprising 25 (22.73%) cases.

**Table 2:** Hysterectomy type and indication (N = 110)

Hysterectomy type	Indication	Number of cases (N)	Percentage (%)
Total abdominal hysterectomy (TAH) with unilateral or bilateral salpingo-oophorectomy	Fibroid	33	30
	Dysfunctional uterine bleeding (DUB)	25	22.73
	Ovarian mass	18	16.37
	Cervical fibroid	3	2.73
Vaginal hysterectomy	Uterovaginal prolapse	31	28.19

**Figure 1:**
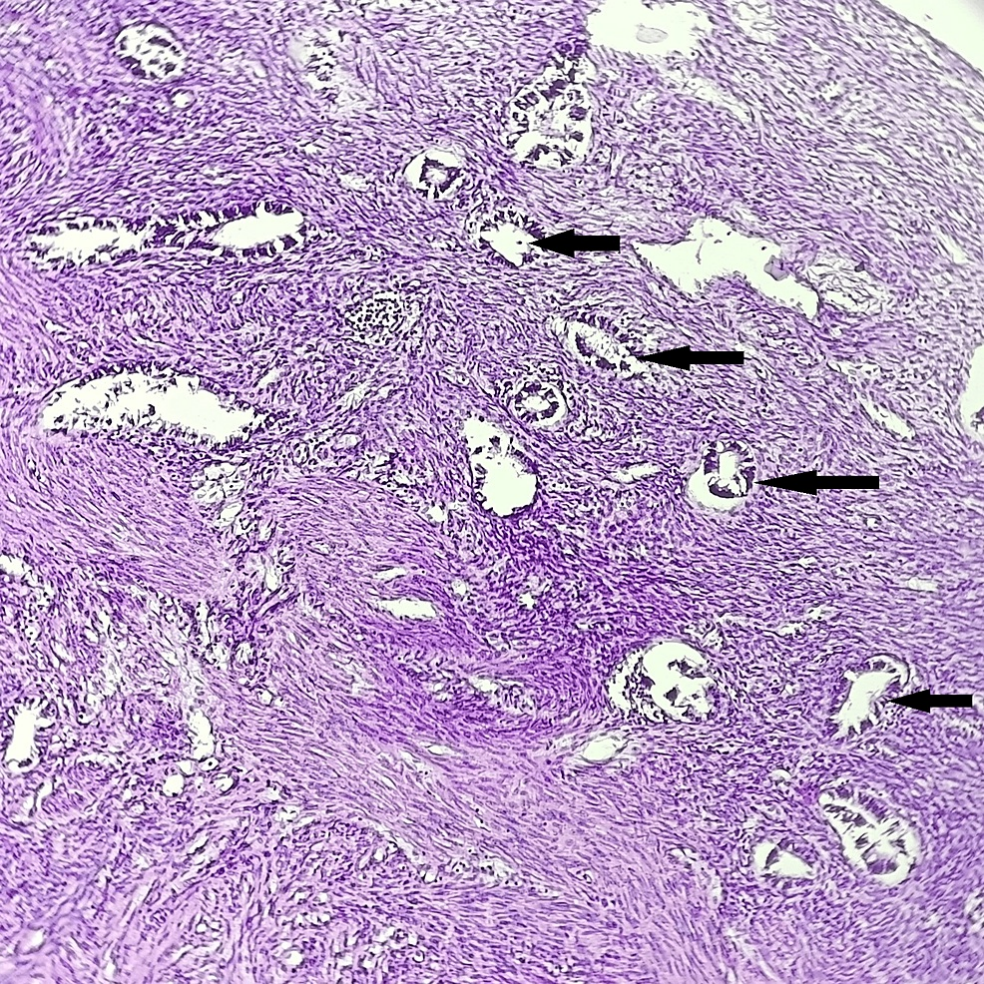
Histopathological photomicrograph showing the proliferative phase of endometrium (H&E, 100X) Black arrows show endometrial glands in the proliferative phase.

Table 3 shows the distribution of histological findings of the endometrium. The most common finding was the endometrium of the proliferative phase in 43 (39.09%) cases (Figure 1). The atrophic endometrium was then more frequently associated with uterovaginal prolapse, seen in 35 (31.82%) cases. Secretory phase endometrium was observed in 21 cases (19.10 %). In two (1.82%) cases, the endometrium showed a polyp. In seven (6.37%) cases, simple endometrial hyperplasia was observed, and in one (0.91%), atypia-related endometrial hyperplasia was noted. Out of 110 cases, a single case (0.91%) was diagnosed as endometrial endometrioid carcinoma, which on microscopic examination, showed a back-to-back arrangement of endometrial glands with cytological dysplasia along with stromal invasion.

**Table 3:** Distribution of histological findings of endometrium (N=110)

Histopathological diagnosis	Number of cases (N)	Percentage (%)
Proliferative phase	43	39.09
Secretory phase	21	19.10
Endometrial hyperplasia without atypia	7	6.37
Endometrial hyperplasia with atypia	1	0.91
Endometrial polyp	2	1.82
Atrophic endometrium	35	31.82
Endometrial carcinoma	1	0.91

**Figure 2:**
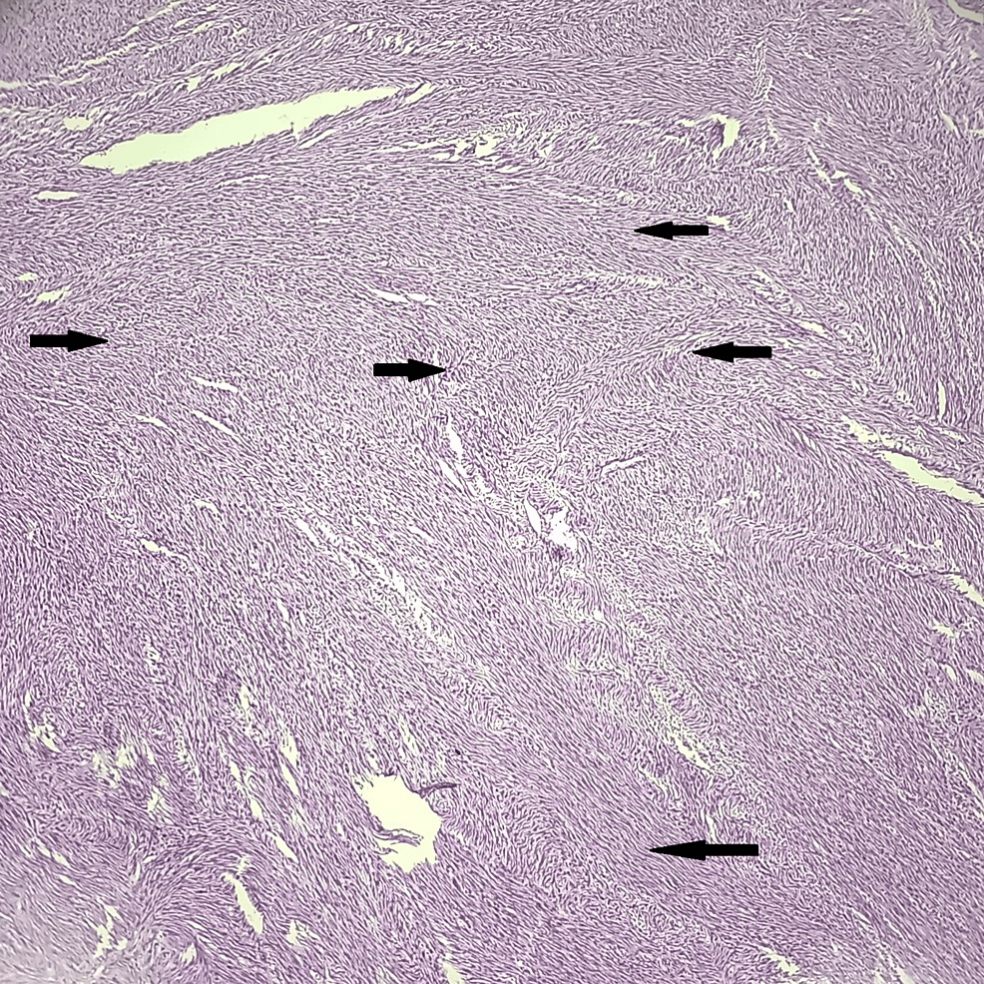
Histopathological photomicrograph showing leiomyoma of uterus (H&E, 100X) Black arrows show interlacing fascicles of smooth muscle cells.

Table 4 illustrates the distribution of myometrial lesions, among which leiomyoma was the most common histopathological finding in 52 cases (47.28 %). Microscopy revealed well-defined tumors composed of oval to spindle-shaped cells with elongated blunt-ended nuclei and a modest amount of eosinophilic cytoplasm. The cells were arranged in the form of interlacing fascicles and bundles (Figure 2). Some leiomyomas exhibited secondary modifications such as hyaline degeneration and myxoid degeneration. The next most common finding was adenomyosis, which was observed in 23 (20.91%) cases. Few cases had both leiomyoma and adenomyosis, seen in 20 (18.19%).

**Table 4:** Distribution of myometrial lesions diagnosed on histopathology (N=110)

Histopathological diagnosis	Number of cases (N)	Percentage (%)
Leiomyoma	52	47.28
Adenomyosis	23	20.91
Leiomyoma + adenomyosis	20	18.19
Normal histology	21	19.10

**Figure 3:**
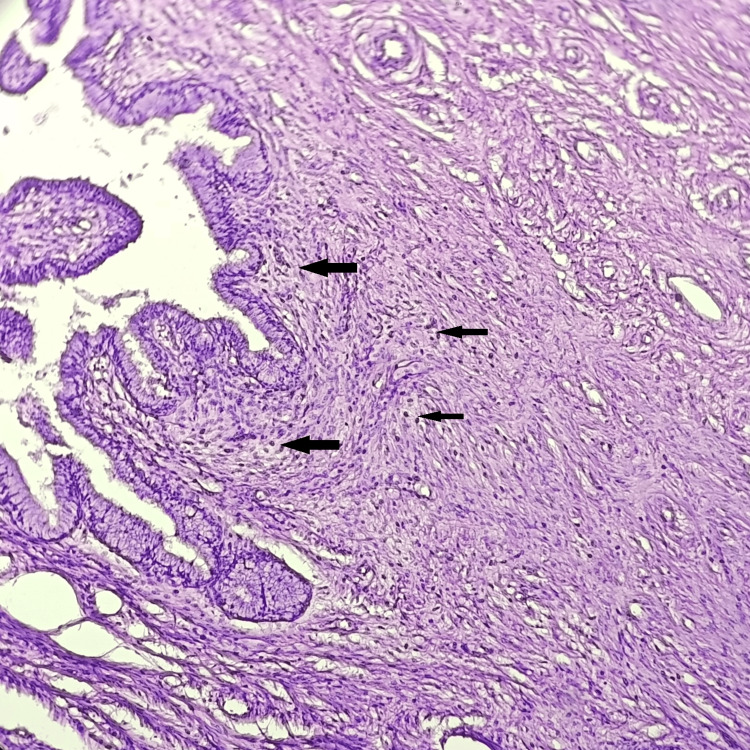
Histopathological photomicrograph showing chronic cervicitis (H&E, 100X) Black arrows show diffuse subepithelial inflammatory infiltrate consisting of lymphocytes.

As shown in Table 5, chronic cervicitis was the most common cervical lesion, comprising 85 (77.28%) cases (Figure 3). Chronic cervicitis with squamous metaplasia was observed in 13 patients (11.82 %). Six (5.46%) cases were papillary endocervicitis and three (2.73%) cases were cervical fibroids.

**Table 5:** Distribution of cervical lesions diagnosed on histopathology (N = 110)

Histopathological diagnosis	Number of cases (N)	Percentage (%)
Chronic cervicitis	85	77.28
Chronic cervicitis with squamous metaplasia	13	11.82
Papillary endocervicitis	6	5.46
Cervical fibroid	3	2.73
Normal histology	3	2.73

**Figure 4:**
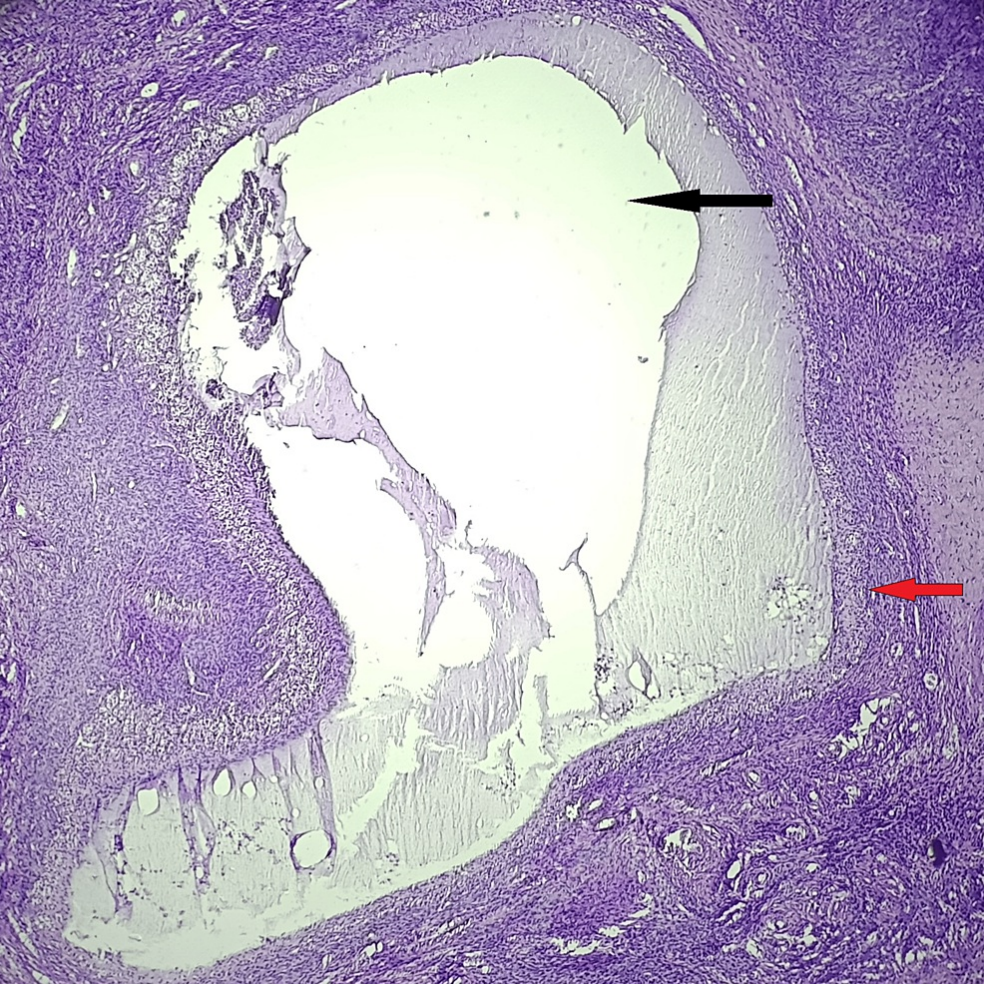
Histopathological photomicrograph showing follicular cyst of ovary (H&E, 100X) The black arrow shows the follicular cyst and the red arrow shows the cyst wall lined by several layers of cells.

Table 6 shows that in the present study, most of the cases (72 of 110) showed normal ovarian histology. There were 28 (25.46%) cases of non-neoplastic lesions and 10 (9.10%) cases of neoplastic lesions in the ovaries. Non-neoplastic lesions included follicular cysts, the most common finding observed in 22 (20%) cases (Figure 4), followed by luteal cysts in 6 (5.46%). Neoplastic lesions included serous cystadenoma as the most common finding in seven (6.37%) cases, followed by one (0.91%) case each of mature teratoma, mucinous cystadenoma, and mucinous cystadenocarcinoma.

**Table 6:** Distribution of ovarian lesions diagnosed on histopathology (N = 110)

Histopathological diagnosis	Number of cases (N)	Percentage (%)
Follicular cyst	22	20
Luteal cyst	6	5.46
Serous cystadenoma	7	6.37
Mucinous cystadenoma	1	0.91
Mucinous cystadenocarcinoma	1	0.91
Mature teratoma	1	0.91
Normal histology	72	65.46

The correlation between the preoperative clinical diagnosis and the histopathological diagnosis is shown in Table 7. In 110 patients, a preoperative clinical diagnosis was available. In most cases, ranging from 70% to 100%, the final histopathological diagnosis supports the preoperative clinical diagnosis. In the current study, a total of two cases of malignant tumors were observed, one case of endometrial carcinoma and one case of mucinous cystadenocarcinoma.

**Table 7:** Correlation between pre-operative clinical diagnosis and histopathological diagnosis (110 cases had pre-operative clinical diagnosis available)

Pre-operative diagnosis	Number of cases (N)	Histopathological diagnosis	
		Number of cases (N)	Percentage (%)
Fibroid	33	28	84.85
Adenomyosis	3	3	100
Serous cystadenoma	10	7	70
Dermoid cyst	1	1	100
Uterovaginal prolapse	31	31	100
Cervical fibroid	3	3	100

## Discussion

Hysterectomy is the most prevalent gynecological operation worldwide. It is an effective procedure to alleviate symptoms and provide patient contentment and offers a permanent solution for many disorders affecting the uterus and adnexa [6]. In this study, the age range of the patients was 25 to 75 years, with a mean age of 50.86 +/- 6.9 years. According to Verma et al. [7] the mean age was 50.1 years, while Adelusola et al. [8] study had a mean age of 49.1 years. In the current study, women between the ages of 35 and 45 years were the most frequently subjected to hysterectomies, which is similar to other studies [9-12]. In the present study, abdominal hysterectomy represented 79 (71.82%) cases and was the surgical procedure performed most frequently, while vaginal hysterectomy accounted for 31 cases (28.19%). In a study by MacKenzie et al. [13], abdominal hysterectomy was preferred in 79% of the cases and vaginal hysterectomy in 17% of the cases. Studies by Sachin et al. [14], Pandey et al. [15], Sujatha et al. [16], and Gupta et al. [17] revealed that the most common hysterectomy procedure was total abdominal hysterectomy. Data from the United Kingdom reveal that abdominal hysterectomy procedures are five to six times more common than vaginal hysterectomy procedures. In a study by Pandya et al. [18], vaginal hysterectomy was the surgical procedure most commonly used in comparison to abdominal hysterectomy.

In our study, fibroids were the most frequent indication of hysterectomy, followed by uterovaginal prolapse, abnormal menstrual cycles, and abdominal masses. According to a study conducted in the United States by Broder et al. [19], fibroids (60%) and prolapse (11%) were the two most common indications. Similar results were found in studies conducted by Jandial [20] and Ullah et al. [21]. Even according to studies by Butt et al. [22], Tiwana et al. [23], Abe et al. [24], and Leung et al. [25], uterine fibroid was the most common indication of hysterectomy. In a study conducted by Verma et al. [7] in Uttar Pradesh, India, uterovaginal prolapse (37.5%) and fibroid uterus (25.6%) were shown to be the most common indications. However, a study by Canadian researchers Toma et al. [26] found that dysfunctional uterine bleeding was the most common indication, followed by uterine fibroid.

The proliferative endometrium (39.7%), which is frequently associated with pathological lesions such as fibroids and adenomyosis, was the most frequent endometrial lesion identified in the present study, followed by the atrophic endometrium (32%), which was frequently observed in postmenopausal women with uterovaginal prolapse. This finding is similar to that of Patil et al. [27], in which the proliferative phase endometrium was the most common endometrial lesion, followed by the atrophic endometrium. Atrophic endometrium was the most prevalent endometrial pathology identified in the study by Kleebkaow et al. [28], who estimated its frequency to be 3.8%. However, Awale et al. [29] observed a greater frequency of atrophic endometrium in their investigation, which was 26.53%.

In our study, leiomyoma was shown to be more frequent than adenomyosis, which has also been observed in studies by Neelgund et al. [30] and Khurshid et al. [31]. In our study, the most frequent incidental finding, chronic cervicitis, was observed in 77.28% of the patients. According to the studies done by Patil et al. [27], Talukder et al. [32], and Khunte et al. [33], the most frequent finding among cervical lesions is chronic cervicitis. The most frequent ovarian lesion observed was a simple follicular cyst, which is consistent with other previous studies by Nausheen et al. [3], Pandey et al. [15], and Perveen et al. [34]. The most frequent benign tumor was a simple serous cystadenoma. The mature cystic teratoma and the mucinous cystadenoma had one case each. There was a case of malignant mucinous cystadenocarcinoma. In the current study, a histopathological evaluation of the fallopian tubes revealed no abnormal lesions. Other studies revealed that the most frequent ovarian abnormalities in their studies were cysts with varied morphologies [30,34,35].

Most of the preoperative clinical diagnoses in our study were supported by histopathological reports, with a proportion ranging from 70% to 100%. Jaleel et al. [36] reported findings that are almost identical to ours. The preoperative clinical diagnosis of adenomyosis, dermoid cyst, uterovaginal prolapse, and cervical fibroid shows 100% correlation with histopathological reports. The only limitation of the current study was the lack of follow-up.

## Conclusions

The present study offers a good understanding of the histopathological patterns of lesions in hysterectomy specimens from our institution. The most prevalent uterine pathology is leiomyoma; the most prevalent ovarian lesion is a follicular cyst; and chronic cervicitis is the most frequently found incidental finding in the cervix in hysterectomy specimens. A total of two cases of malignant tumors are noted: one case of endometrial carcinoma and one case of mucinous cystadenocarcinoma of the ovary. Few lesions, including chronic cervicitis and adenomyosis, are discovered purely as incidental findings, although histopathological analysis and clinical diagnoses generally correlate well. To ensure better postoperative management, it is imperative that every hysterectomy specimen, even if it superficially appears to be normal, be subjected to a thorough histopathological examination.
